# Survey data on supply chain improvement and operational competency of oil and gas firms in Nigeria

**DOI:** 10.1016/j.dib.2018.08.150

**Published:** 2018-09-01

**Authors:** Bolanle D. Motilewa

**Affiliations:** Covenant University, Ota, Nigeria

**Keywords:** Supply chain improvement, Operational competency, Corporate social responsibility, Organisational Performance, Developing countries, Human Capital Development

## Abstract

The firm׳s suppliers are in most social responsibility literature considered a branch of the firm׳s stakeholders that may not necessarily benefit directly from the firm׳s social responsibility practices. However recent studies on CSR from the developing country׳s perspective has highlighted the importance that needs to be placed on supply chain improvement. Thus, this article presents data on the effect of supply chain improvement as a construct of corporate social responsibility on operational competency. The study employed a descriptive quantitative research design survey method. The study population consists of 1748 employees from four top oil and gas firms quoted in the Nigerian stock exchange. A sample size of 350 employees were selected. Data was analysed using statistical Package for Social Sciences (SPSS). Regression analysis was employed as the statistical tool of analysis. The field data set is made widely accessible in this article.

## Specification table

**Table:** 

**Subject area**	Business and Management
**More Specific Subject Area**	Supply chain management, Human Capital Development, Corporate Social Responsibility,
**Type of Data**	Tables
**How Data was Acquired**	Survey
**Data Format**	Raw, Filtered and Descriptive
**Experimental Factors**	A simple random sampling technique was used to gather the data
**Experimental features**	The gathered data were based on randomly selected respondents among employees in oil and gas firms operating in Lagos.
**Data source location**	South west, Nigeria
**Data Accessibility**	Data is provided with this article

## Value of data

• The data provided gives an insight on the firms’ involvement in supply chain improvement within the confines of corporate social responsibility in oil and gas firms in Nigeria. Further studies can review this stance in other industries.
• The provided data also shows statistics on CSR from the developing country׳s perspective. Considering the limited available data on CSR that goes beyond philanthropy from the developing country׳s perspective, future studies might consider expanding their investigation into other aspects of CSR beyond philanthropy.
• Considering the limited available data on employees’ perception of the firm׳s commitment to supply chain improvement, as well as lack of data set on this area of corporate social responsibility, the provided data opens up avenue for future studies focused on the creating shared value concept of CSR.

## 1 Data

A total of three hundred and fifty copies of questionnaire were administered to respondents from the top four listed oil and gas firms in Nigeria׳s stock exchange. Table 1 below shows that 22.9% of the population of this study were from Firm 1, 27.3% from Firm 2, 27.8% from Firm 3 and 22% from Firm 4. This clearly shows that each firm for the study was well represented. The demographic characteristics of the respondents are also highlighted in Table 2 below.

**Table 1:** Sample frame for distribution of questionnaire.

**Name of firm**	**Number of employees**	**Percentage of total (%)**		**Questionnaire distributed**
Firm 1	401	401/1748 * 100	22.9	80
Firm 2	477	477/1748 * 100	27.3	96
Firm 3	485	485/1748 * 100	27.8	97
Firm 4	385	385/1748 * 100	22.0	77
**Total**	**1748**		**100**	**350**

**Table 2:** Demographic characteristics of respondents.

**Demographic characteristics**	**Items**	**Oil and Gas Firms**				**Total**
		**Firm 1 (%)**	**Firm 2 (%)**	**Firm 3 (%)**	**Firm 4 (%)**	
**Gender**	Male	38 (49.4)	40 (44.4)	59 (62.8)	57 (76.0)	194 (57.7)
	Female	39 (50.6)	50 (55.6)	35 (37.2)	18 (24.0)	142 (42.3)
**Total**		**77**	**90**	**94**	**75**	**336**
**Age**	Under 25 yrs	–	9 (10.0)	–	11 (14.7)	20 (5.9)
	25–35 yrs	36 (46.7)	52 (57.8)	23 (24.5)	33 (44.0)	144 (42.9)
	36–45 yrs	21 (27.3)	17 (18.9)	67 (71.3)	31 (41.3)	136 (40.5)
	46 yrs +	20 (26.0)	12 (13.3)	4 (4.3)	–	36 (10.7)
**Total**		**77**	**90**	**94**	**75**	**336**
**Length of Service**	Less than 5 yrs	57 (74.0)	51 (56.7)	70 (74.5)	22 (29.3)	200 (59.5)
	5–10 years	20 (26.0)	21 (23.3)	20 (21.3)	26 (34.7)	87 (25.9)
	11–15 years	–	4 (4.4)	4 (4.3)	25 (33.3)	33 (9.8)
	16 yrs and above	–	14 (15.6)	–	2 (2.7)	16 (4.8)
**Total**		**77**	**90**	**94**	**75**	**336**
**Status or Position**	Director	–	–	–	–	–
	Senior Manager	20 (26.0)	9 (10.0)	44 (46.8)	2 (2.7)	75 (22.3)
	Analyst	–	28 (31.1)	17 (18.1)	23 (30.7)	68 (20.2)
	Supervisor	57 (74.0)	8 (8.9)	2 (2.1)	41 (54.7)	108 (32.3)
	Others	–	45 (50.0)	31 (33.0)	9 (12.0)	85 (25.3)
**Total**		**77**	**90**	**94**	**75**	**336**
**Educational Status**	OND/NCE	–	13 (14.4)	–	7 (9.3)	20 (5.9)
	HND/B.Sc.	38 (49.4)	36 (40.0)	60 (63.8)	27 (36.0)	161 (47.9)
	MSc/MBA/MEd	39 (50.6)	26 (28.9)	31 (33.0)	37 (49.3)	133 (39.6)
	Others	–	15 (16.7)	3 (3.2)	4 (5.3)	22 (6.6)
**Total**		**77**	**90**	**94**	**75**	**336**

### 1.1 Statement of test statistics

Given that the correlation co-efficient measures the degree to which two things vary together, this model correlated two variables: supply chain improvement and operational competency.

Table 3 showed the descriptive statistics of supply chain improvement and operational competency for each oil and gas Firm. Respondents from all the sampled firms agreed with all the constructs in this variable. Most of the respondents acknowledged positively to the contribution of firm׳s supply chain improvement on its operational competency. Nonetheless Firm 1(4.461), Firm 4 (4.313) and Firm 2 (4.007) respondents admitted favourably to the statement however, respondents from Firm 3 (3.993) slightly agreed. This indicates that management of Firm 3 needs to develop strategies that will help to facilitate their supply chain improvement.

**Table 3:** Descriptive statistics of variables for each oil and gas firm.

**Descriptive statistics**								
	**Firm 1**		**Firm 2**		**Firm 3**		**Firm 4**	
	**Mean**	**SD**	**Mean**	**SD**	**Mean**	**SD**	**Mean**	**SD**
**Supply Chain Improvement**	4.461	0.380	4.007	0.452	3.993	0.287	4.313	0.454
**Operational Competency**	3.879	0.417	4.086	0.467	3.939	0.315	3.950	0.803
	**Freq = 77**		**Freq = 90**		**Freq = 94**		**Freq = 75**	

^a^Predictors: (Constant), Supply Chain Improvement: *Honesty in contracts with suppliers, dialogue with suppliers, promotion of local suppliers, support of local suppliers, compliance with industry standards*.

^b^Dependent variable: Operational Competency.

The above Table 4 showed the statistical significance of the two variables for each oil and gas firm: supply chain improvement and operational competence using the multiple regression. The statistics presented in the table above under *R* square is called the coefficient of determination and referred to as *R*^2^. The *R* Square tells how much of the variance in the dependent variable (operational competency) is explained by the independent variable (supply chain improvement). The *F* statistic tests the overall significance of the model. In this case, the value for each firm (Firm 1 = 0.920, Firm 2 = 0.601. Firm 3 = 0.510 and Firm 4 = 0.579) is expressed as a percentage, this means that the independent variable (supply chain improvement) explains Firm 1 (92%), Firm 2 (60.1%), Firm 3 (51%) and Firm 4 (57.9%) of the variance in operational competency (Table 5).

**Table 4:** Model characteristics for each firm.

**Firm 1**			**Firm 2**			**Firm 3**			**Firm 4**		
***r***	***r***^**2**^	**Sig.**	***r***	***r***^**2**^	**Sig.**	***r***	***r***^**2**^	**Sig.**	***r***	***r***^**2**^	**Sig.**
0.959^a^	0.920	0.000^b^	0.775^a^	0.601	0.000^b^	0.714^a^	0.510	0.000^b^	0.761^a^	0.579	0.000^b^
*F* = 864.158			*F* = 132.341			*F* = 95.857			*F* = 100.284		

a Dependent Variable: Operational Competency.

b Predictors: (Constant), Supply Chain Improvement

**Table 5:** Model summary.

**Model summary**			
Multiple *R*	*R* Square	Adjusted *R* square	Apparent prediction error
0.754	0.569	0.560	0.431

^c^Predictors: (Constant), Supply Chain Improvement: *Honesty in contracts with suppliers, dialogue with suppliers, promotion of local suppliers, support of local suppliers, compliance with industry standards.*

^a^Dependent variable: Operational Competency.

The above Table 6 showed the statistical significance of the two variables of supply chain improvement and operational competency using the categorical regression.

**Table 6:** Model summary (ANOVA^a^).

**ANOVA**					
	Sum of squares	d*f*	Mean square	*F*	Sig.
Regression	191.065	13	14.697	32.653	0.000
Residual	144.935	322	0.450		
Total	336.000	335			

^b^Predictors: (Constant), Supply chain improvement.

a Dependent Variable: Operational Competency.

Table 7 shows the combined influence of the independent variables (training honesty in contracts with suppliers, dialogue with suppliers, promotion of local suppliers, support of local suppliers, industry standards) on operational competency (the dependent variable) of the firms.

**Table 7:** Model summary (coefficients)^a^.

	Standardised coefficients		d*f*	*F*	Sig.
	Beta	Bootstrap (1000) estimate of Std. Error			
The firm has a policy to ensure honesty and quality in all its contracts with its suppliers	0.499	0.122	2	16.814	0.000
The firm has a process to ensure effective feedback, consultation and dialogue with suppliers	0.180	0.092	2	3.818	0.023
The firm encourages the use of local content	0.396	0.075	2	27.596	0.000
The firm has a commitment to providing technical capacities and assistance to local suppliers	0.490	0.078	4	39.267	0.000
The firm actively advocates new industry standards for sustainable development	0.195	0.136	3	2.032	0.009

Dependent Variable: Operational Competency.

The result in Table 8 shows the staff opinion on the potential of the firm׳s commitment to supply chain improvement in facilitating the operational competency of oil and gas firms, and it reveals that honest and quality in contracts with their suppliers is a major predictor of operational competency, which has the highest beta value of beta = 0.499, *p* < .005, Sig. 0.000, than other variables: providing technical capacities and assistance to local suppliers (support of local suppliers) scaled (beta = 0.490, *p* < .005, Sig. 0.000), promotion of local suppliers scaled (beta = 0.144, *p <* .005, Sig. 0.004). Statistically, this means that honesty in contracts with suppliers makes the strongest unique contribution in influencing operational competence.

**Table 8:** Total number of employees.

	**Firm 1**	**Firm 2**	**Firm 3**	**Firm 4**	**Total**
Number of Employees	401	477	485	385	1748

## 2 Experimental design, materials and methods

The data presented a quantitative research based on a descriptive research design to assess the effect of supply chain improvement on operational competency of oil and gas firms within the confines of corporate social responsibility and from a developing country׳s perspective. Survey method was considered appropriate for data gathering.

The population of the study consists of the stakeholders of four (4) top oil and gas firms listed on the Nigerian stock exchange. The choice of these firms is in support of previous studies [1], [2] where it was statistically proffered that the study of corporate social responsibility is best situated in firms with top financial performance, as indicated by high stock price, which invariably means that the firm can carry out its economic obligations, and as such has resources to deal with social problems [3], [4], [5], [6]. In total, there are one thousand, seven hundred and forty eight (1748) employees in all four firms. 350 employees were judiciously selected to partake in this research [7]. Data were collected from these organizations using an adapted researcher made questionnaire. A proportional analysis was conducted to determine the number of copies of the questionnaire to be distributed to the individual firms. The questionnaire is in two sections A and B. Section A contains background questions, section B consists of questions that are specific to the data provided, that is supply chain improvement and operational competency.

The data was coded and keyed into the statistical package for social sciences (SPSS) version 22. Data was described using inferential statistical tests involving multiple regression analysis.

The researchers ascertained that respondents were well informed about the background and the purpose of the research. Every respondent was entitled to the opportunity to stay anonymous and their responses treated with utmost confidentiality. Permission was obtained from the appropriate authorities in the firms where copies of the research instruments were distributed.
