# Supplementary material for: Survey data on supply chain improvement and operational competency of oil and gas firms in Nigeria
Source: Data Brief. 2018 Sep 1;20:1073–8. doi: 10.1016/j.dib.2018.08.150 (PMC6140360; doi:10.1016/j.dib.2018.08.150)
Supplement: Supplementary file 2 — Supplementary material [file mmc2.docx]

# RESEARCH QUESTIONNAIRE ON SUPPLY CHAIN IMPROVEMENT AND OPERATIONAL COMPETENCY

Dear respondent,

This questionnaire aims to get data on the above topic. I kindly ask for your voluntary co-operation in filling out this questionnaire.

Please note that the exercise is strictly for academic purpose and the information provided will be kept in strict confidence. Response to this questionnaire is absolutely voluntary and kindly note that you can decide at any point in time not to take part in this survey.

Thank you for your anticipated support and co-operation

**(Researcher)**

**SECTION A: DEMOGRAPHIC DATA (Please tick whichever is applicable)**

1. Gender: Male Female
2. Age:
3. Under 25 years b. 25 – 35 years

c. 36 – 45 years d. 46 years and above

1. Length of service in the Oil and Gas Sector
2. Less than 5 years b. 5 – 10 years
3. 11 – 15 years d. 16 years and above
4. Position in the organisation
5. Director b. Senior Manager

c. Supervisor d. Analyst

d. Others, please specify ………………….

1. Please select the category you feel most appropriately describes your unit in the organisation
   1. Engineering b. Administrative

c. Finance and Accounts d. Analyst

Others, please specify ………………….

1. Educational qualification
2. OND/NCE b. HND/BSc.

c. MSc/MBA/M.Ed. d. Others

1. Number of years working in the company:
2. Less than 5 years b. 5 – 10 years
3. 11 – 15 years d. 16 years and above

**Supply chain Improvement and Operational Competency**

| **S/No** | **Item** | **SA** | **A** | **U** | **D** | **SD** |
| --- | --- | --- | --- | --- | --- | --- |
| 1 | The firm has a policy to ensure honesty and quality in all its contracts with its suppliers |  |  |  |  |  |
| 2 | The firm has a process to ensure effective feedback, consultation and dialogue with suppliers |  |  |  |  |  |
| 3 | The firm encourages the formation of networks or cooperatives of small suppliers |  |  |  |  |  |
| 4 | The firm is committed to provide technical capacities and assistance to local suppliers |  |  |  |  |  |
| 5 | The firm actively advocates new industry standards for sustainable development |  |  |  |  |  |
| 6 | The firm’s encouragement of networks of small suppliers has helped reduce its inventory time (time needed to get supplies) |  |  |  |  |  |
| 7 | The firm’s process of effective feedback, consultation and dialogue with suppliers helps forge community relations |  |  |  |  |  |
| 8 | The firm’s commitment to providing technical assistance to local suppliers ensures it has access to quality supplies |  |  |  |  |  |
| 9 | The firm’s policy to ensure honesty and quality in all its contracts with its suppliers helps it get reliable and prompt information from its suppliers |  |  |  |  |  |
| 10 | The firm’s compliance with industry standards forges a good relationship with the industry generally |  |  |  |  |  |
