# Supplementary material for: Survey data on supply chain improvement and operational competency of oil and gas firms in Nigeria
Source: Data Brief. 2018 Sep 1;20:1073–8. doi: 10.1016/j.dib.2018.08.150 (PMC6140360; doi:10.1016/j.dib.2018.08.150)
Supplement: Supplementary file 3 — Supplementary material. [file mmc3.docx]

**COVER LETTER**

This manuscript describes original work and is not under consideration by any other journal. All authors approved the manuscript and this submission for your consideration for publication in Data in Brief. Please find the enclosed manuscript entitled **Survey Data on Supply Chain Improvement and Operational Competency of Oil and Gas Firms in Nigeria**

Bolanle D. Motilewa

[Bolanle.motilewa@covenantuniversity.edu.ng](mailto:Bolanle.motilewa@covenantuniversity.edu.ng)

**CA: Bolanle D. Motilewa**

**[bolanle.motilewa@covenantuniversity.edu.ng](mailto:bolanle.motilewa@covenantuniversity.edu.ng)**

**COVENANT UINVERSITY**
